# What Intentions and Interesting Information Can Attract Consumers to Scan QR Code While Buying Eggs?

**DOI:** 10.3390/foods11091259

**Published:** 2022-04-27

**Authors:** Shang-Ho Yang, Huong Thi Thu Phan, Chi-Ming Hsieh, Tzu-Ning Li

**Affiliations:** 1 Graduate Institute of Bio-Industry Management, National Chung Hsing University, No. 145 Xingda Road, South District, Taichung City 40227, Taiwan; bruce.yang@nchu.edu.tw; 2 International Master Program of Agriculture, National Chung Hsing University, Taichung City 40227, Taiwan; rosyphan@smail.nchu.edu.tw; 3 International Bachelor Program of Agribusiness, National Chung Hsing University, Taichung City 40227, Taiwan; 4 Department of Applied Science of Living, Chinese Culture University, 55, Hwa-Kang Road, Yang-Ming-Shan, Taipei 11114, Taiwan

**Keywords:** QR Code, traditional market, supermarket, egg

## Abstract

A Quick Response Code (QR Code) aims to provide accurate and traceable information to consumers wanting to verify the quality of agri-food products. This study aimed to investigate the experiences and intentions of scanning QR Code in traditional markets and supermarkets. Furthermore, the types of egg information in the QR Code were explored to identify consumer interests when purchasing eggs. The empirical data were collected from 1112 valid responses throughout Taiwan from July to September, 2020. The Logit, Probit models, and the Bivariate Probit model were used to examine the data. Results showed that shoppers’ propensity to scan QR Code revealed a significant difference between traditional markets and supermarkets, i.e., supermarket shoppers having higher a propensity to scan a QR Code. Of the 10 types of potential egg information in the QR Code, over half of respondents said that the production certificate label and inspection information were the top reasons that they would be interested in scanning a QR Code. This was particularly the case for homemakers aged between 51 and 60 years old and those who had scanned QR Code before and would like to pursue more egg information. Since the egg producers have resisted joining the traceability system, the implication of this study provides very practical strategies for government, policy makers, and producers in Taiwan.

## 1. Introduction

In recent years, concerns about food safety issues have evolved in consumers’ health consciousness. Products with traceability and transparent information are becoming more important for consumers wanting to obtain more information about their food products [1,2]. In Taiwan, the consciousness regarding egg products has emerged in the last few years, which has decreased consumers’ trust about egg safety [3]. On the other hand, egg and its related products were found to be easily contaminated by various pathogens at any stage of processing; even those eggs with fresh and uncracked shells still could lead to a potential foodborne disease due to the Salmonella infection [4]. Moreover, eggs play a vital role in the daily diet of consumers and provide a convenient and high-quality protein for consumers’ health [5]. In order to help consumers to avoid hazardous or harmful food products and enhance consumers trust, a QR Code label attached on packages would perform a significant role in conveying additional information and detailing more accurate processing information [6,7].

The QR Code was originated in Japan in 1994 for the use of tracking parts consumed in automobile manufacture. Later, QR code were launched in Taiwan in 2005, getting utilized and popularized in food industries [8]. The QR Code is widely used in many determinations, such as promotion, mobile payment, surveying, product information [9,10,11]. Mostly, the application of QR Code in the agricultural and food industries is for the purpose of product traceability. Labels with a QR Code printed on the packaging of agri-food products are presented as a tool that can transmit the product information from producers to consumers [7,12]. Moreover, consumers are expecting a QR Code to be provided for more crucial information about food products [13] (such as its brand, origin, naturalness, packaging, pricing, nutrition, safety, ingredients, sustainable plantation method, food processing method, and environmental effects) or marketing messages [14] (such as an incentive offer, discounts, and coupons). However, information is still limited as to which kinds of product and marketing information should be discoverable via the QR Code and how these information impact consumers’ decisions.

In the past, there was no traceability labeling on the crates of non-packed eggs, and it was impossible to trace back the information source of egg farms whenever any issues related to eggs were discovered, as was reported by the Council of Agriculture (COA) in Taiwan in 2014. Hence, accurate information of egg production features is needed to regain consumers’ trust. With such attention on the enhancement of egg sanitation, quality, and safety for egg consumption, the COA has intensely promoted QR Code labeling, serving since 2015 as a channel between producers and consumers who would like to request the traceable information. In Taiwan, QR Code labeling for food traceability has been classified broadly across the country [15]. Consumers are increasing their knowledge awareness via QR Code to trace food products they are interested in, are expecting to acquire more information via their QR Code, and may hope to utilize QR Code if needed [8]. In order to achieve domestic food safety, the COA has released the label of the Traceable Agriculture Products (TAP), which contains a QR Code labeling that can obtain traceable information [16].

According to Taiwan Poultry and Products (2016), traditional market vendors commonly sell eggs counting by weight, while several local grocery stores sell counting by quantity amount [17]. When eggs are sold that are randomly picked and counted by weight, the food safety risk may potentially exist. Furthermore, vendors in traditional markets or local grocery stores are often challenged to provide more product information if the supply chain system does not support to it. Therefore, it may create opportunities for consumers to explore different types of eggs in supermarkets or hypermarkets where consumers normally can find eggs with different appeals, i.e., animal welfare, free range, organic, food safety certified, traceability, QR Code, etc. In general, eggs in convenience stores, supermarkets, and hypermarkets are mostly washed, graded, and packaged in plastic or cardboard egg boxes. The modalities in supermarkets particularly can be distinguished from those eggs sold in traditional markets or local grocery stores. This may imply that the request of product information in different markets, i.e., traditional markets and supermarkets, may not be the same regarding the concern of food safety.

A traditional market is commonly a face-to-face transaction for foods and other daily goods, while supermarkets are presenting for the westernized retail provision with larger quantities of foods and goods [18]. In Taiwan, traditional markets are majorly positioned in open spaces or under a roof, and there are 709 traditional markets settled publicly and privately across the country, according to the Taiwan Ministry of Economic Affair (2022) [19]. Traditional markets play an important role as most food products are sold from local areas with flexible pricing, various sources of local food products, freshness offers, independent sellers, custom services, and bargaining experience. However, in traditional markets there are still deficiencies in product labeling, nutritional, and traceability information [20,21]. On the other hand, the store settings of supermarkets and hypermarkets are categorized in various classifications that can shorten shopping time and increase the chance of revisiting shopping [22]. As of 2021, there are about 186 hypermarket outlets (i.e., Carrefour and Costco) [23] and 2286 supermarkets (i.e., Simple Mart, Welcome, PX-Mart, etc.) [24] in Taiwan. It shows that buyers can quite equally choose which types of markets they would like to purchase their eggs from. Therefore, the consumer behavior between traditional markets and supermarkets or hypermarkets may not be the same.

Furthermore, consumer attitudes towards food safety may differ according to socio-economic factors and personal preferences [25]. Thus, the research objectives of this study are to examine consumer intentions in using QR Code to access information, and which kinds of information they would be interested in receiving from QR Code and in traditional markets and supermarkets. Thus, our three objectives are as follows:

(1) to identify shoppers’ experiences of scanning QR Code and the reason why they do not want to scan;
(2) to examine whether the shopping environment (traditional markets versus supermarket) impacts consumers’ propensity to scan QR Code;
(3) to analyze what social demographics and shopping background would influence buyers’ intentions to scan QR Code;
(4) to explore what kinds of egg information shoppers are interested in receiving by scanning QR Code.

## 2. Materials and Methods

### 2.1. Empirical Models

#### 2.1.1. The Logit and Probit Model

The propensity for scanning QR Code can be related to shoppers’ social demographic factors and shopping habit factors. Furthermore, the probability of shoppers scanning the QR Code is the main question. A common approach is to adopt the Logit and Probit model to analyze the prediction of the probability for those who have scanned QR Code in the past. This application is in correspondence with many previous studies [26,27]. Pourhoseingholi et al. (2008) investigated the relationship between social demographic factors and attributes related to the probability of gastrointestinal cancer [26]. Scarpato et al. (2017) determined some of the main factors that influence the likelihood of consumers being attentive to food safety based on the variables of consumers shopping habits and food safety attributes [27]. Thus, the Logit and Probit models are adopted in this study and presented as the following:

(1) $$Y_{i}=\alpha +\beta x+\varepsilon$$

The dependent variable, ***Y_i_***, is a binary response, which indicates whether a respondent has scanned a QR Code before or not:

$$Y=\{\begin{array}{c} 0\;if\;no\\ 1\;if\;yes \end{array}$$

As in Equation (1), the ***Y_i_*** is unobservable, **x** is independent variables, and *β* is the regression coefficient that associates with unknown parameters, *ε*, which denotes the error term in a normal distribution. Thus, ***Y_i_*** here can be identified with two alternative results: a respondent has scanned a QR Code before (***Y_i_*** = 1) and a consumer has not scanned a QR Code before (***Y_i_*** = 0).

The Logit model or logistic regression model can specify Equation (2), which is a Cumulative Distribution Function (CDF) of the logistic distribution (i.e., Λ(*α*′*β*)), while the Probit model can specify a conditional probability, which can be expressed by Equation (3), if F(**x′***β*) is the standard normal distribution. Both are with the predicted probabilities limited between 0 to 1.

The probability of the Logit model can be expressed as Equation (2):

(2) $$F\left(x^{\prime }\beta \right)=\Lambda \left(x^{\prime }\beta \right)=\frac{e^{x^{\prime }\beta }}{1+e^{x^{\prime }\beta }}=\frac{\exp\left(x^{\prime }\beta \right)}{1+\exp\left(x^{\prime }\beta \right)}$$

The probability of the Probit model can be specified as Equation (3):

(3) $$F\left(x^{\prime }\beta \right)=\Phi (x^{\prime }\beta )=\int_{-\infty }^{x^{\prime }\beta }\phi \left(z\right)dz$$

The model coefficients are calculated by using the maximum likelihood method. We maximize the log-likelihood as Equation (4):

log = yF(**x**′*β*) + (1 − y)[1 − F(**x**′*β*)] (4)

We can further calculate the marginal effects (M.E.) to obtain a probability explanation of independent variables. The marginal effects indicate the proportional change in the probability of dependent variable (y = 1) based on 1 unit change of an independent variable **x**. Marginal effects for the Logit model can be expressed as Equation (5):

(5) $$\partial p/\partial x_{j}=\Lambda \left(x^{\prime }\beta \right)\left[1-\Lambda \left(x^{\prime }\beta \right)\right]\beta_{j}=\frac{e^{x^{\prime }\beta }}{{\left(1+e^{x^{\prime }\beta }\right)}^{2}}\beta_{j}$$

Marginal effects for the Probit model can be exhibited as Equation (6):

(6) $$\partial p/\partial x_{j}=\phi \left(x^{\prime }\beta \right)\beta_{j}$$

If the estimated parameters of the independent variables and marginal effects indicate a significant level at the same time for both the Logit and Probit models, the independent variable will be treated as a significant factor and it will be further explained.

#### 2.1.2. Bivariate Probit Model

Since two binary outcome models (i.e., Probit models used in traditional markets and supermarkets) are adopted in this study, the estimated outcomes may be correlated. Thus, this study further applies the Bivariate Probit Model to examine whether the individual decision of scanning a QR Code may be correlated between traditional markets and supermarkets. Regarding the application of the Bivariate Probit models, many studies [28,29] have adopted this method. Castillo-Manzano (2010) employed the Bivariate Probit model to analyze whether consumers would like to purchase food/beverages at airport stores and airport catering [28], while Torres et al. (2017) adopted the Bivariate Probit model to investigate whether the marketing strategies should be implanted in the organic certification decision or not [29]. Thus, the unobserved latent variables in this study can be presented as Equation (7):

(7) $$y_{1}^{*}=x_{1}^{\prime }\beta_{1\;}+e_{1}\;y_{2}^{*}=x_{2}^{\prime }\beta_{2\;}+e_{2}$$

where $y_{1}^{*}$ and $y_{2}^{*}$ represent unobserved latent variables that can be explained by the observed independent variables. The $x_{1}^{\prime }\;\mathrm{and}\;x_{2}^{\prime }$ denote the observed independent variables, which are the vector of variables for explaining the dependent variables $y_{1}^{*}$ and $y_{2}^{*}$. The coefficients, i.e., $\beta_{1}$ and $\beta_{2}$, are estimated parameters for the explanatory variables, and the $e_{1}$ and $e_{2}$ are the error terms of the two models. If the error terms are correlated to each other, it means that these two separate Probit models are endogenously determined. The correlation parameter ρ will show a significance outcome if there exists an exogeneity in the error terms [30]. It implies that shoppers’ scanning QR Code behavior at traditional markets and supermarkets may be correlated. Thus, a Bivariate Probit model should be adopted; otherwise, it can just run two different Probit models.

For the explanatory variables, a model specification of variables was contained and based on social demographics, shopping background, and interested information.

$$y_{1}^{*}=\{\begin{array}{c} 1,\;if\;one\;tends\;to\;scan\;QR\;code\;in\;traditional\;markets\\ 0,\;ifone\;does\;not\;tends\;to\;scan\;QR\;code\;in\;traditional\;markets \end{array}\;y_{2}^{*}=\{\begin{array}{c} 1,\;if\;one\;tends\;to\;scan\;QR\;code\;in\;supermarkets\\ 0,\;ifone\;does\;not\;tends\;to\;scan\;QR\;code\;in\;supermarkets \end{array}$$

The estimation and probabilities of the Bivariate Probit model can be exhibited as Equation (8):

(8) $$P(y_{1},y_{2}|x_{1},\;x_{2})=\Phi \left[q_{i1}\beta_{1}x_{i1}^{\prime },\;q_{i2}\beta_{2}x_{i2}^{\prime }\rho \right]$$

The coefficient estimation can be calculated as the following log-likelihood approach in Equation (9):

(9) $$\mathrm{Log}\;L=\sum_{i}lnP(y_{1}y_{2}|x_{1},x_{2})$$

The Bivariate Probit model can further estimate the marginal effects and predicted values, which are calculated similarly by the Binary Probit model. Marginal effect is a joint probability that can be calculated and expressed in Equation (10):

(10) $$E[y_{1}|y_{2}=1,x_{1},x_{2}]=\frac{P[y_{1}=1|y_{2}=1,x_{1},x_{2},\rho ]\;}{P[y_{2}=1|x_{1}]}$$

In order to examine a correlation of the independent variables in this study, this study uses the Variance Inflation Factor (VIF) to detect the issue of multicollinearity in regression analysis. The formula of the VIF can be revealed by Equation (11).

(11) $$\mathrm{VIF}=\frac{1}{1-R_{i}^{2}}$$

### 2.2. Questionnaire Design and Distribution

Normally, fresh eggs buyers are those who are in charge of cooking and grocery shopping in a family [21]. In order to efficiently recruit respondents who fitted into these backgrounds, two screening questions are designed in the questionnaire (also shown in the Supplementary Material Questionnaire S1). Furthermore, this study adopts a lucky draw event to incentivize potential egg buyers to fill out our survey. A total of 150 7-Eleven gift cards valued $100 NTD per each are provided in the lucky draw. Respondents are notified before they fill out the survey. These two screening questions are: (1) “Are you the person who normally does the grocery shopping at home?”; and (2) “Have you purchased fresh raw eggs within the past half-year?”. Thus, those who have never done the grocery shopping or bought eggs within the past half-year will be eliminated from the SurveyMonkey questionnaire system. The sampling approaches involve sharing the survey links through a Facebook page under the advertisement promotion, sharing through the Line group message, and sharing through the public area, such as parks, train stations and near elementary school during pick-up time. The staff of our research team is trained to kindly check respondents’ willingness. If respondents are not fitting the screening questions, these respondents are simply not invited.

The sample data collection was conducted from June to September in 2020. Although there was concern about the COVID-19 issue, the market condition was not affected and did not display differences from the non-COVID-19 period. A total of 1555 responses participated to fill out the survey. After eliminating the non-qualified respondents, a total of 1112 responses were valid to be analyzed in this study. In summary, the sample respondents of the social demographic variables are defined in Table 1. In order to make sure our sample data reaches a good level of representation, female (about 75% out of total respondents), age (about 40 years old), education year (about 15 years), family number (about 3 people in a household), and main buyer (about 52%) show slightly higher figures when compared with government reports [31]. However, it is still a good indicator when compared to previous studies [20,21], since our study focuses on those who are the major buyers for food products in a family. In particular, respondents aged over 41 years old occupy about 49%, which is an important indicator for analyzing who are the potential buyers for using QR Code during food shopping. Moreover, older people are difficult to encourage to use QR Code, so the sample data present a reasonable representation in this study.

Table 1 is categorized into three groups described with the mean value. The demographic background shows that 75% of respondents are women and 25% of respondents are from 41 to 50 years old. Respondents, on average, earned a high education level about at bachelor’s degree. The monthly income of a household averagely amounts to about $62,570 NTD. There were about three to four family members in their household. Regarding the occupation categories, respondents who are homemakers totaled about 14%, who worked in the manufacturing sector about 10%, and who worked in the service industry about 19%. In the shopping background section, the main buyers are the majority of respondents. On average, respondents are frequently cooking at home almost eight times a week, and this implies that most respondents cook every day. In particular, about 92% of respondents normally spend more than 15 min on their shop at the grocery stores. Respondents usually spend about 2.25 times that to shop at supermarkets every month, about 1.79 times at traditional markets, about 1.11 times at hypermarkets, and approximately 0.44 times at farmers markets. The last section is the list of 10 kinds of egg information that would be interesting to scan QR Code for. It shows that respondents (about 58%) are most likely to scan QR Code if the Production certificate label is included in the egg information.

## 3. Results and Discussion

Following the methodology set-up and the reasonably representative data set, questions as to why respondents have or have not scanned QR Code in the past are asked. In particular, the reasons why egg shoppers did not scan QR Code before were questioned and further analyzed.

### 3.1. The Reasons Why Consumers Do Not Scan QR Code

**Figure 1:**
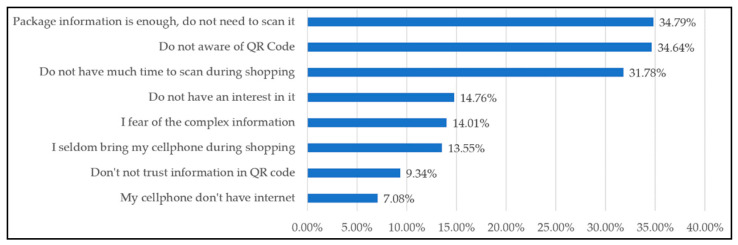
The Reasons Why Respondents Have Not Scanned QR Code Before.

The rate of those who are experienced in scanning a QR Code in the past is lower in comparison with those who have never scanned it (or unsure how to scan) is approximated (57.91%). This implies that more than half of respondents have not scanned QR Code before. Thus, it is important to explore reasons for those who have not scanned QR Code before. According to Figure 1, the primary three reasons for consumers’ limited scanning were “package information is enough, do not need to scan it” (34.79%), followed by “Do not be aware of QR Code” (34.64%), and “Do not have much time to scan during shopping” (31.78%). These findings are likely similar to a previous study that indicates that the greatest reason is that most consumers are not aware of QR Code’s usefulness (40.49%), consumers do not feel interested in QR Code (12.77%), and that the QR Code provided on the back of product are not safe (20.81%) [32].

### 3.2. Who Have Scanned QR Code for Purchasing

Table 2 presents the results of the Logit and Probit models with the characteristics of social demographics and shopping background of experienced respondents who had scanned a QR code before. These two models obtained statically significant outcomes by the presence of an Adjust R^2^ test and a Wald (χ^2^) test, which meant these results were valid. As in Table 2, it should be marked that the socio-demographic aspects do not affect those who have experience in scanning a QR Code for buying. Instead, the shopping background aspect was shown to be positive and statistically significant at many variables for those who are main buyers of food shopping, who are frequently cooking at home, and who are visiting farmers’ markets, supermarkets, hypermarkets, revealing that these variables had an impact on the experience of scanning QR code before. This implies that shopping background would be the most major factor in scanning QR Code, not social demographic factors.

In order to reach an approximate model between the Logit and Probit results, this can be achieved by choosing the minimum value of the Akaike Information Criterion (AIC) and Bayesian Information Criterion (BIC) [21]. Hence, this study selected the result of the Probit model as the outcome for major characteristics of respondents who have scanned QR Code for their purchasing. Additionally, the VIF numerical value showed at 1.28 which meant this model did not exist as a multicollinearity issue among the entire independent variables in this study.

### 3.3. Consumers’ Propensity to Scan QR Code in Traditional Markets and Supermarkets

**Figure 2:**
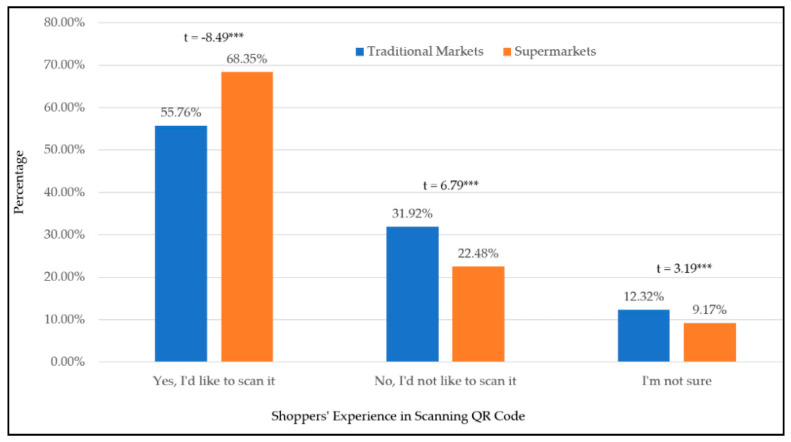
The Percentage of Shoppers Who Have Scanned QR Code in Traditional Markets and Supermarkets. *** *p* < 0.001.

Subsequently, respondents were asked their intentions for scanning QR Code in traditional markets and supermarkets for egg purchase. Figure 2 shows that the intentional comparison regarding whether egg shoppers would like to scan QR Code in traditional markets and supermarkets. Expressly, the percentage of supermarket shoppers who would like to scan for egg purchase is significantly higher than those in traditional markets following a t-test outcome. Moreover, the unintentional group means those respondents would not scan QR Code in either traditional markets or supermarkets. The percentage of respondents who are unlikely to scan QR Code in traditional markets is significantly higher than those in supermarkets following a t-test outcome. Although some respondents show hesitancy in scanning QR Code in markets, the percentage of traditional market shoppers is significantly higher than those in supermarkets following a t-test outcome. Thus, the results in Figure 2 indicated that shoppers in traditional markets were more reluctant to scan QR Code.

To further analyze the major characteristics of the intentional group, this study used the Bivariate Probit model to indicate the influence of socio-demographic variables, shopping background, and the interested QR Code information for respondents to scan in traditional markets and supermarkets. Foremost, as seen in Table 2, the Wald test of Rho (ρ) value was determined at 230.9, which was statistically significantly different from zero (*p* = 0.000), implying that that these two Probit models for the intentions of scanning QR Code in traditional markets and supermarkets were correlated to each other and that the Bivariate Probit model was needed in this data examination. In addition, the Wald (χ^2^) test value was presented at 345.932, which shows a statistically significant difference from the Bivariate Probit model. Table 3 shows that the test results were qualified and justified to figure out the characteristics of traditional and supermarket consumers’ intentions in scanning QR Code for egg purchase.

In the section of socio-demographic characteristics, the female and manufacture variables displayed a positive and significant sign in both markets. This shows that females had a potential impact on the intention to scan QR Code in traditional markets (9.1%) and supermarkets (9.6%) based on the marginal effect when compared to male shoppers. Specifically, the female participants who had the manufacturing industry occupation showed a higher proportion of scanning QR Code in the traditional market (13.8%) than those in the supermarkets (11.5%). However, those whose occupation related to services tended somewhat (5.4%) to scan QR Code at supermarkets only. From the perspective of the shopping background, it was significant that 5.2% of main buyers intended to scan QR Code in supermarkets when purchasing eggs, and it was also significant that participants who frequently cooked at home intended to scan QR Code in both markets. However, egg shoppers who spent more than 15 min in supermarkets were more likely to scan QR Code than those who spent less than 15 min in shopping. Consumers who were more often visiting traditional markets would have a 1.9% higher chance of scanning QR Code when compared to those were less frequently visiting. On the other hand, egg shoppers who were more often visiting supermarkets would have a 1.5% higher frequency of scanning QR Code compared to those were less frequently visiting. One significant finding identified was that the experienced respondents tended to scan QR Code for their eggs purchasing in traditional markets (17.6%) and supermarkets (21.1%). This study links to previous study [22] in that some demographic variables and shopping preferences were quite effective in distinguishing traditional market buyers from supermarket buyers in Taiwan.

Regarding the interested information section, these 10 kinds of egg information could possibly incentivize consumers to scan QR Code when purchasing eggs. For those consumers who are interested in the producer information, production certificate label, inspection information, production video record, and carbon footprint, they would tend to scan QR Code when receiving the above information in both of the mentioned markets. Consumers caring about traceability information are likely to scan QR Code in supermarkets. On the other hand, consumers are unlikely to scan QR Code if they are only interested in discounts in traditional markets, or recipe recommendations in supermarkets.

### 3.4. What Information Makes Consumers Want to Scan QR Code

**Figure 3:**
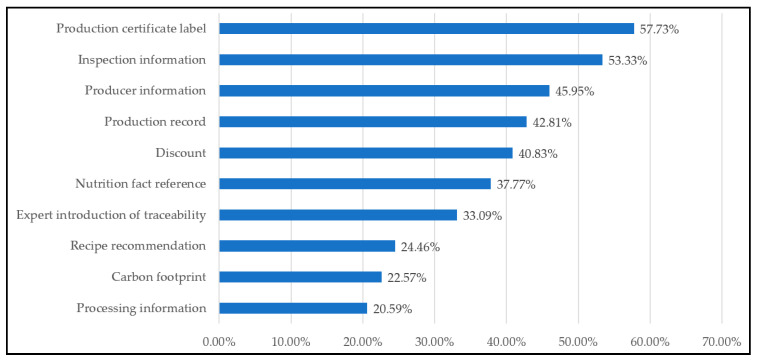
The Egg Information That Consumers Would Be Interested to Scan QR Code for.

Since egg information is attractive to many shoppers, the frequency of each piece of egg information was ranked and exhibited in Figure 3. In Figure 3, the top three pieces of information that drive consumers to scan QR Code include production certificate label (57.73%), inspection information (53.33%), and producer information (45.95); the least preferred information includes recipe recommendation (24.46%), carbon footprint (22.57%), and processing information (20.59%). This study found that the majority of respondents were most likely concerned about whether traceability eggs had carried any certificate label and information associated with the feeding of the animal and the use of pesticides, while they were less likely to be concerned about the information of processing, such as production technical process, product processing factory information, information on additives and quality control standards. The above finding is somewhat consistent with prior research regarding consumers’ awareness of food safety and involved two aspects, which are inspection information and quality certification [33,34]. However, there is some inconsistency between this study and previous study [35], which showed that process and input traceability are what has the greatest impact on consumers’ trust regarding food safety when purchasing a food product.

Table 4 presents the results of analyzing these ten kinds of interested information attributes based on consumers’ social demographics and shopping habits to determine which factors would affect the probability of consumers’ characteristics for scanning QR Code when purchasing eggs.

The estimation results of the Logit model for the probabilities of the interested information that could incentivize consumers to scan QR Code when purchasing eggs are documented in Table 4. All of the attributes displayed significance with the Wald χ^2^ test. Thus, it is justified that the model specification in each piece of interested information reasonably fit. Regarding the factors of the interested information, several significant findings are provided and discussed. Firstly, the socio-demographic segment indicated that the female coefficients were negative and significant at the attributes of producer information, production video record, and processing information, inferring that male shoppers are more interested in these kinds of information. However, female shoppers were interested in the recipe recommendation provided through QR Code when buying eggs. On the other hand, the age category showed that the group of respondents aged 41 to 50 years old had a negative impact on the interested information of discount, nutrition references, recipe recommendation, and processing information, while this age group were positively interested in producer information, such as name, address, phone number, etc. These results link to previous studies’ findings that female and older consumers tend to seek more food labels and information when buying food products [35]; likewise, as age goes up, consumers are more likely to investigate product information and peruse food labels [36].

Secondly, the education variable had a positive and significant impact on the information of production video record and expert introduction of traceability instead of discount, which carried a negative and significant sign. This means that highly educated respondents are more interested in the egg information provided rather than the discount, which is consistent with previous findings that higher-educated consumers look more for labeling information [37,38]. Meanwhile, the high-income levels did not affect such behavior since the high-income households responded favorably to information for carbon footprint. The family number coefficient could be explained by the fact that, if the family has more members, they have higher intentions to scan QR Codes as long as the discount code is provided; however, they do not seem interested in the information of production record and carbon footprint. The homemakers expressed a high potential interest in the information of producer information, inspection information, and expert introduction of traceability, but were unfavorable to the nutrition reference. Moreover, those who had occupations of manufacturing industries were more likely interested in the production video record.

Thirdly, the test results of the variable of shopping background indicated that the main buyer variable had a negative impact on the preferred information of production certificate label and carbon footprint. In general, the spending time for food shopping does not exceed 15 min and those shoppers are unlikely to scan QR Code because of the production certificate labels. Consumers who frequently visit traditional markets are unlikely to scan QR Code because of the inspection information. Meanwhile, consumers frequently visiting supermarkets are likely interested in the information of the production certification label, but they are uninterested in the expert information of traceability. Consumers with past experience of scanning QR Code are likely to scan it on account of much preferred information, except the discount and recipe recommendation. It could be explained that consumers recognize that the product quality could exceed the value of discount and recipe services, and thus these factors did attract consumers to scan [38].

## 4. Conclusions and Implication

In order to enhance the food safety consciousness in the egg industry, the Taiwanese government has intensively encouraged farmers and food companies to adopt the egg traceability system from farms to consumers through the presence of QR Code, while there were many voices that suggest consumer ignorance regarding scanning QR Code hinders the promotion of the traceability system. Thus, it is important to investigate consumers’ intention to scan QR Code for egg products in traditional markets and supermarkets. In particular, what kinds of information provoke interest and how to increase the chance for consumers to scan QR Code are investigated in this study. A total of 1112 valid respondents were collected and analyzed. Results show that only about 42% of respondents had scanned QR Code before, which corresponds to the concern of negative voices from egg industry. It implies that government or policy makers would need more understanding about: (1) why egg shoppers do not scan; (2) which market venues would need more promotion to scan; (3) what kinds of egg information consumers would be interested in scanning when they are buying eggs.

Since most egg shoppers are found to be the majority not scanning QR Code, the reasons for not scanning QR Code are explored. The results reveal that the top three reasons are particularly different from other reasons. The top three reasons include: (1) enough information from package, so not scanning; (2) not aware of QR Code; and (3) not enough time to scan. These outcomes may correspond to previous study [33], which showed that some marketplaces can provide a scanning device to increase frequency of scanning QR Code. However, egg buyers often may only have a little time for grocery shopping. Even providing a scanning device may be not much help. The results of scanning QR Code experience show that most factors involve consumer shopping background, not social demographic factors. In particular, those who are the main buyers, frequently cooking at home, and frequently visit farmers markets, supermarkets, and hypermarkets have the highest chance of having scanned QR Code before.

Regarding the scanning behavior in traditional markets and supermarkets, it shows that supermarket shoppers are likely to scan QR Code than those in traditional markets. It definitely points out that more promotions and incentives to scan QR Code in traditional markets are needed. However, regarding the incentive to scan QR Code in traditional markets, the discount information in the QR Code would not increase the chance to scan at all. Although supermarket shoppers are interested in more information in QR Code, the information of recipe recommendation was not preferred. The results in this study would definitely help marketers to set up the necessary information for their shoppers in traditional markets and supermarkets.

Concerning the interesting traceability information, we realized that respondents are mostly attracted by the information of the production certificate label, inspection information, and producer information. Despite the processing information, carbon footprint and recipe recommendation are the least favorite pieces of information for consumers to scan QR Code. Respondents characterized as male, aged between 41 and 60 years old, and with past experience of scanning QR Code are those who are potentially interested in scanning QR Code for the producer information. Furthermore, respondents with higher levels of education are more likely interested in the detailed information of traceability and production records for scanning QR Code when buying eggs. Another remarkable finding is that those who work in the manufacturing sector and high-level education tend to be incentivized to scan QR Code for the production video records information. However, this finding is not in line with a previous study that shows that QR Code scanning behavior by incentive offered, access to discounts, deals, or coupons are the highest motivating factors [21]. Egg producers or practitioners are expected to accommodate consumers’ expectations by matching their preferred traceability information among target segments.

In sum, the findings of this study play an important role for policymakers, marketing planners, or related egg industries when considering a strategic plan in the egg production business as well as traceability information development in traditional markets and supermarkets in Taiwan. Although this study concentrated on examining traditional and supermarket consumers’ intentions, the interesting information for scanning QR Code for egg purchase, and potentially interesting traceability information, some limitations (i.e., experience of buying eggs, frequency of paying attention to the labels, food safety perception, and trust) may require further exploration

## Figures and Tables

**Table 1:** Demographic Samples and Variable Definition.

Variables	Description	Mean
A. Demographics Background		
Female	DV = 1, if the respondent is female, 0 o/w	0.75
Age (41–50 years)	DV = 1, if the respondent is from 41 to 50 years old, 0 o/w	0.25
Age (51–60 years)	DV = 1, if the respondent is from 51 to 60 years old, 0 o/w	0.18
Age (60 years above)	DV = 1, if the respondent is from 61 years old to above, 0 o/w	0.06
Education	CV; years of education	15.45
Family income	CV; the household’s income monthly ($1000 NTD)	62.57
Family number	CV; numbers of family number	3.51
Homemaker	DV = 1, if the respondent is a household, 0 o/w	0.14
Retired	DV = 1, if the respondent is retired, 0 o/w	0.06
Service	DV = 1, if the respondent works in a service industry, 0 o/w	0.19
Manufacture	DV = 1, if the respondent works in manufacturing, 0 o/w	0.10
B. Shopping Background		
Main buyer	DV = 1, if the respondent is a primary food shopper for family, 0 o/w	0.52
Cooking Frequency	CV; numbers of cooking-at-home frequency in a week	7.76
Time-spending (≤15 min)	DV = 1; if the respondent usually spends not more than 15 min for their food shopping, 0 o/w	0.08
Visit-farmers markets frequency	CV; frequency of visiting farmers markets for purchasing eggs in the last 1 month	0.44
Visit-traditional markets frequency	CV; frequency of visiting traditional markets for purchasing eggs in the last 1 month	1.79
Visit-supermarkets frequency	CV; frequency of visiting supermarkets for purchasing eggs in the last 1 month	2.25
Visit-hypermarket frequency	CV; frequency of visiting hypermarkets for purchasing eggs in the last 1 month	1.11
Have-scanned-QRcode	DV = 1, if the respondent has scanned QR Code before, 0 o/w	0.42
C. Interested Information		
Producer information	DV = 1, if the information of producers is provided through QR Code scanning, the respondent will tend to scan the QR Code, 0 o/w	0.46
Expert introduction of traceability	DV = 1, if the expert introduction of traceability is provided through QR Code scanning, the respondent will tend to scan the QR Code, 0 o/w	0.33
Discount	DV = 1, if the discount is provided through QR Code scanning, the respondent will tend to scan the QR Code, 0 o/w	0.41
Recipe recommendation	DV = 1, if the recipe recommendation is provided through QR Code scanning, the respondent will tend to scan the QR Code, 0 o/w	0.24
Production certificate label	DV = 1, if the production certificate label is provided through QR Code scanning, the respondent will tend to scan the QR Code, 0 o/w	0.58
Inspection information	DV = 1, if the inspection information is provided through QR Code scanning, the respondent will tend to scan the QR Code, 0 o/w	0.53
Production video record	DV = 1, if the production video record is provided through QR Code scanning, the respondent will tend to scan the QR Code, 0 o/w	0.43
Processing information	DV = 1, if the processing information is provided through QR Code scanning, the respondent will tend to scan the QR Code, 0 o/w	0.21
Nutrition reference	DV = 1, if the nutrition fact reference is provided through QR Code scanning, the respondent will tend to scan the QR Code, 0 o/w	0.38
Carbon footprint	DV = 1, if the information of carbon footprint is provided through QR Code scanning, the respondent will tend to scan the QR Code, 0 o/w	0.23

Note: (DV) and (CV) represent the dummy and continuous variables, respectively; the o/w represents otherwise.

**Table 2:** The Logit and Probit Model among Experienced Respondents.

Variables	The Logit Model		The Probit Model	
	Coef.	M.E.	Coef.	M.E.
Social demographics				
Female	−0.069	−0.016	−0.042	−0.016
Age (41–50 years)	0.094	0.022	0.057	0.022
Age (51–60 years)	0.088	0.021	0.053	0.020
Age (60 years above)	−0.274	−0.063	−0.167	−0.062
Education	0.035	0.008	0.022	0.008
Family income	0.001	0.000	0.000	0.000
Family number	0.074	0.017	0.047	0.018
Homemaker	−0.245	−0.057	−0.153	−0.057
Retired	−0.356	−0.081	−0.226	−0.084
Service	0.243	0.058	0.151	0.058
Manufacture	0.136	0.032	0.086	0.033
Shopping background				
Main buyer	0.239 *	0.056 *	0.150 *	0.057 *
Cook-at-home frequency	0.022 *	0.005 *	0.013 *	0.005 *
Time-spending (≤15 min)	−0.229	−0.053	−0.144	−0.054
Visit-farmers-markets frequency	0.115 **	0.027 **	0.072 **	0.027 **
Visit-traditional-markets frequency	−0.046	−0.011	−0.029	−0.011
Visit-supermarkets frequency	0.070 **	0.016 **	0.044 **	0.017 **
Visit-hypermarket frequency	0.086 **	0.020 **	0.053 **	0.020 **
Constant	−1.647 ***		−1.027 ***	
Numbers of obs.	1112		1112	
Adjusted R^2^	0.028		0.028	
Wald (χ^2^)	41.12 ***		42.46 ***	
Log-Likelihood	−735.959		−735.837	
AIC	1509.92		1509.68	
BIC	1605.18		1604.94	
VIF	1.28		1.28	

Note: ***, **, * means significance at 1%, 5%, and 10%, respectively. Coef. denotes the coefficient, and M.E. present marginal effect.

**Table 3:** The Results of Bivariate Probit Model in Analyzing Shoppers’ Propensity to Scan QR Code in Two Different Markets.

Bivariate Probit Model	Traditional Markets		Supermarkets	
	Coef.	M.E.	Coef.	M.E.
Social Demographics				
Female	0.289 ***	0.091 ***	0.302 ***	0.096 ***
Age (41–50 years)	0.108	0.040	−0.036	−0.017
Age (51–60 years)	0.203	0.068	0.035	0.004
Age (60 yrs above)	0.000	0.003	0.055	0.010
Education	−0.024	−0.008	−0.021	−0.008
Family income	−0.002	−0.001	−0.001	0.000
Family number	0.032	0.009	−0.020	−0.006
Homemaker	−0.037	−0.014	−0.135	−0.055
Retired	−0.224	−0.069	−0.227	−0.083
Service	0.096	0.034	0.197 *	0.054 *
Manufacture	0.443 ***	0.138 ***	0.435 ***	0.115 ***
Shopping Background				
Main buyer	0.051	0.016	0.199 **	0.052 **
Cook-at-home frequency	0.029 ***	0.010 ***	0.021 **	0.006 **
Time-spending (≤15 min)	−0.078	−0.032	−0.294 **	−0.086 **
Visit-traditional-markets frequency	0.057 **	0.019 **	0.032	0.011
Visit-supermarkets frequency	0.012	0.003	0.046 **	0.015 **
Have-scanned-QRcode	0.520 ***	0.175 ***	0.706 ***	0.211 ***
The Interested Information				
Producer information	0.396 ***	0.132 ***	0.469 ***	0.139 ***
Expert introduction of traceability	0.123	0.039	0.264 ***	0.083 ***
Discount	−0.154 *	−0.051 *	−0.144	−0.044
Recipe recommendation	−0.068	−0.026	−0.185 *	−0.042 *
Production certificate label	0.475 ***	0.167 ***	0.429 ***	0.123 ***
Inspection information	0.146 *	0.051 *	0.298 ***	0.080 ***
Production video record	0.247 ***	0.077 ***	0.427 ***	0.126 ***
Processing information	0.043	0.016	−0.033	−0.001
Nutrition reference	−0.001	−0.001	−0.001	0.011
Carbon footprint	0.192 *	0.061 *	0.277 **	0.077 **
Constants	−1.042 **		−0.761	
Adjusted R^2^	0.162		0.235	
Log-Likelihood	−639.993		−531.181	
Number of observation	1112	Mean dependent var		0.683
SD dependent var	0.465	Wald (χ^2^)		345.932
Wald test of Rho (ρ)	230.9 ***	Prob > χ^2^		0.000

Note: ***, **, * means significance at 1%, 5%, and 10%, respectively. Coef. denotes the coefficient, and M.E. present marginal effect.

**Table 4:** The Results of the Logit Model Estimates for What Kinds of Egg Information Are Interested in Scanning QR Code by Shoppers.

	Attributes	1. Prod Certificate Label		2. Inspection Information		3. Producer Information		4. Production Video Record		5. Discount		6. Nutrition Reference		7. Expert Introduction of Traceability		8. Recipe Recommendation		9.Carbon Footprint		10. Processing Info	
Variables		Coef.	M.E.	Coef.	M.E.	Coef.	M.E.	Coef.	M.E.	Coef.	M.E.	Coef.	M.E.	Coef.	M.E.	Coef.	M.E.	Coef.	M.E.	Coef.	M.E.
Social demographics																					
Female		−0.038	−0.009	0.042	0.010	−0.562 ***	−0.135 ***	−0.272 *	−0.064 *	0.200	0.046	0.074	0.017	0.110	0.024	0.561 ***	0.093 ***	0.204	0.033	−0.302 *	−0.050 *
Age (41–50 years)		0.185	0.043	0.195	0.047	0.441 ***	0.105 ***	0.008	0.002	−0.567 ***	−0.126 ***	−0.345 **	−0.077 **	0.168	0.037	−0.359 *	−0.061 **	−0.272	−0.044	−0.392 **	−0.059 **
Age (51–60 years)		0.709 ***	0.158 ***	0.491 ***	0.117 ***	0.580 ***	0.139 ***	0.006	0.001	−1.021 ***	−0.216 ***	−0.542 ***	−0.118 ***	0.398 **	0.089 *	−0.511 **	−0.084 **	−0.343	−0.054	−0.570 **	−0.081 ***
Age (60 years above)		0.534	0.118	0.528	0.124	0.001	0.000	−0.527	−0.117	−1.110 ***	−0.220 ***	−0.517	−0.111	0.232	0.051	−1.324 ***	−0.172 ***	−0.306	−0.047	−0.377	−0.054
Education		−0.035	−0.008	0.052	0.013	0.040	0.009	0.127 ***	0.029 ***	−0.059 *	−0.014 *	−0.051	−0.012	0.064 *	0.014 *	−0.026	−0.005	0.035	0.006	0.038	0.006
Family income		−0.002	−0.000	0.001	0.000	−0.000	−0.000	0.001	0.000	0.002	0.001	0.002	0.000	−0.001	−0.000	0.001	0.001	0.006 ***	0.001 ***	−0.001	−0.000
Family number		0.062	0.014	−0.014	−0.003	0.008	0.002	−0.104 **	−0.024 **	0.103 **	0.024 **	−0.017	−0.004	−0.038	−0.008	−0.014	−0.002	−0.216 ***	−0.036 ***	−0.002	−0.000
Homemaker		0.388 *	0.088 *	0.393 *	0.094 *	0.150	0.036	0.024	0.006	−0.251	−0.057	−0.548 **	−0.119 ***	0.589 ***	0.135 ***	0.036	0.006	−0.028	−0.005	−0.173	−0.027
Retired		−0.041	−0.009	−0.172	−0.042	0.571	0.136	0.249	0.058	0.488	0.114	−0.105	−0.024	0.093	0.020	0.281	0.053	−0.699	−0.098 *	0.072	0.012
Service		−0.000	−0.000	−0.041	−0.010	−0.134	−0.032	0.158	0.037	−0.099	−0.023	0.112	0.026	0.183	0.040	−0.472 **	−0.078 ***	−0.231	−0.037	−0.169	−0.026
Manufacture		0.250	0.057	0.280	0.067	−0.068	−0.016	0.605 ***	0.142 ***	−0.190	−0.043	0.070	0.016	−0.004	−0.001	−0.423	−0.069	−0.596 **	−0.087 **	0.236	0.039
Shopping Background																					
Main buyer		−0.285 **	−0.066 *	−0.151	−0.036	0.050	0.012	−0.196	−0.046	0.106	0.024	−0.095	−0.022	0.028	0.006	−0.028	−0.005	−0.352 **	−0.059 **	−0.118	−0.019
Cook-at-home frequency		−0.017	−0.004	−0.002	−0.000	0.001	0.000	0.021	0.005	−0.019	−0.004	−0.009	−0.002	0.019	0.004	0.005	0.001	0.008	0.001	0.031 **	0.005 **
Time-spending (≤15 min)		−0.449 *	−0.106 *	−0.090	−0.022	−0.021	−0.005	−0.310	−0.070	0.063	0.015	0.174	0.041	0.017	0.004	−0.332	−0.055	0.372	0.066	−0.415	−0.060
Visit-traditional-markets frequency		0.015	0.004	−0.050 *	−0.012 *	0.013	0.003	−0.040	−0.009	−0.003	−0.001	0.025	0.006	0.019	0.004	−0.014	−0.002	−0.03	−0.005	−0.028	−0.004
Visit-supermarkets frequency		0.057 *	0.013 *	0.024	0.006	−0.006	−0.001	0.038	0.009	−0.008	−0.002	0.015	0.004	−0.060 *	−0.013 *	−0.015	−0.003	−0.042	−0.007	−0.020	−0.003
Have-scanned-QRcode		0.473 ***	0.110 ***	0.358 ***	0.087 ***	0.413 ***	0.099 ***	0.322 **	0.075 **	−0.436 ***	−0.100 ***	0.130	0.030	0.360 ***	0.079 ***	−0.305 **	−0.054 **	0.299 **	0.050 *	0.299 **	0.048 *
Constant		0.463		−0.939		−0.814		−2.013 ***		0.602		0.423		−2.071 ***		−0.663		−1.201		−1.622 **	
Adjusted R^2^		0.035		0.018		0.031		0.043		0.038		0.020		0.021		0.032		0.048		0.028	
Log-Likelihood		−730.867		−754.492		−743.22		−726.275		−723.078		−722.571		−690.814		−598.629		−565.154		−549.460	
Wald χ^2^		48.17		26.84		45.62		61.55		54.42		27.65		28.71		40.45		51.63		33.42	
Prob > χ^2^		0.0001		0.0605		0.0002		0.0000		0.0000		0.0492		0.0372		0.0011		0.0000		0.0100	

Note: ***, **, * means significance at 1%, 5%, and 10%, respectively. Coef. denotes the coefficient, and M.E. present marginal effect.

## Data Availability

The data are not publicly available due to the data belong to the project funders.

## Supplementary Materials

The following are available online at https://www.mdpi.com/article/10.3390/foods11091259/s1, Questionnaire S1: The Questionnaire Used in this Study.
