# Supplementary material for: What Intentions and Interesting Information Can Attract Consumers to Scan QR Code While Buying Eggs?
Source: Foods. 2022 Apr 27;11(9):1259. doi: 10.3390/foods11091259 (PMC9102952; doi:10.3390/foods11091259)
Supplement: Supplementary file 1 [file foods-11-01259-s001.zip › foods-1669170-supplementary.pdf]

*Supplementary Materials*

**Questionnaire S1.** The Questionnaire Used in this Study.

## 雞蛋購買行為問卷調查 Eggs Purchasing Behavior Survey (Foods journal)

### 感謝您的參與 Thanks for your participation.

Have you bought eggs before? We want to understand your opiniions about fresh eggs. We kindly invite you to fill our survey. It may take you about 5 minutes. This survey is only used for academic research, so please feel free to answer it and please do not leave any your own privacy information. For those who can complete our survey, we will provide a lucky draw of the 7-11 gift card (valued $100). We will draw out 150 lucky respondents, so please help us to share our survey if you can. If you have any question, please feel free to contact us at MIRS Facebook page or huongptt0305@gmail.com

NCHU-IMPA Ms. Phan
Professor: Dr. Yang

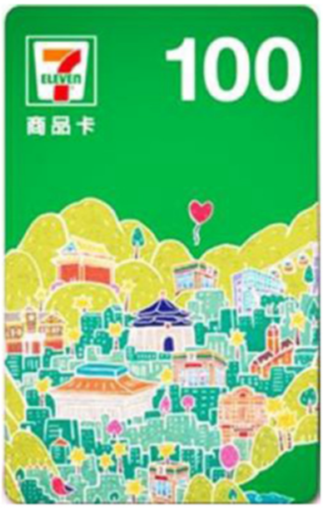


*** 1. 您是家中主要負責買菜的人嗎?**
**Are you the person who normally does the grocery shopping at home?**

- ◯ 是，大部分時候 Yes, most of time
- ◯ 是，有時候 或 買某些部分 Yes, sometimes
- ◯ 不是，完全沒有 No, not at all

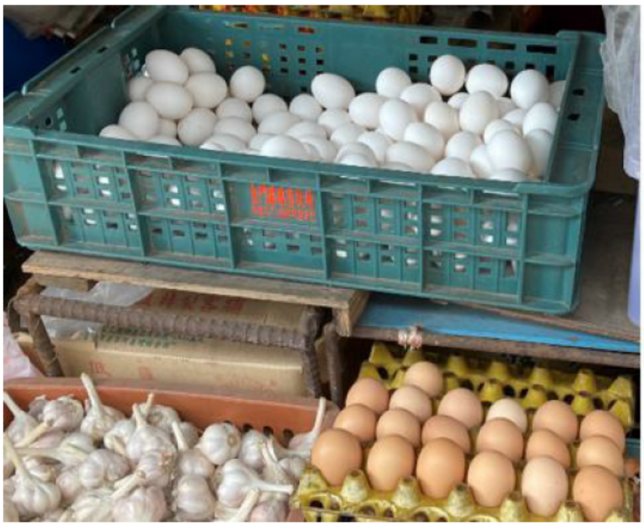

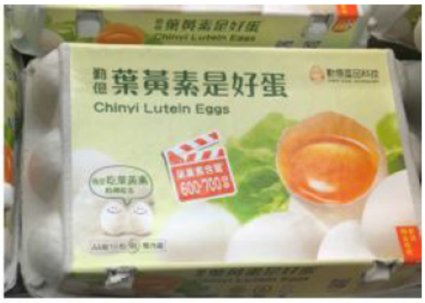



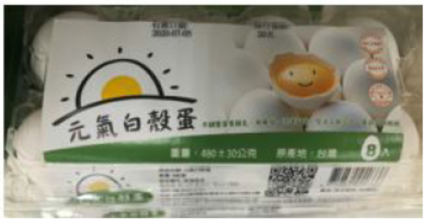



*** 2. 請問您最近半年內有沒有買過生鮮雞蛋 (如上圖)?**

**Have you purchased raw eggs in the past half year (Like the above picture)?**

◯ 有，家裡的雞蛋 都是我在買的
Yes, most of situations I am responsible to buy eggs

◯ 有，有時候 會幫忙買蛋
Yes, occasionally I help to buy eggs

◯ 沒有，從沒有買過
No, never buy before

## 第1.1部分

*** 3. 在一週當中，以早、中、晚餐為基準，您家裡大約開伙幾次?**
**How often do your family cook at home in a week?**

- ◯ 0 次 time
- ◯ 1-3次 times
- ◯ 4-6次 times
- ◯ 7-9次 times
- ◯ 10-12次 times
- ◯ 13-15次 times
- ◯ 16次以上 times & up

*** 4. 在一週當中，以早、中、晚餐為基準，您自己外食次數大約幾次?**
**How often do you eat out in a week?**

- ◯ 0 次 time
- ◯ 1-3次 times
- ◯ 4-6次 times
- ◯ 7-9次 times
- ◯ 10-12次 times
- ◯ 13-15次 times
- ◯ 16次以上 times & up

*** 5. 平常在 購買食材 時，您會花多少時間 完成？**
**During the grocery shopping, how much time you would usually need?**

- ◯ 15分鐘以下 15 mins or below
- ◯ 16至30分鐘 Between 16 to 30 mins
- ◯ 31至45分鐘之間 Between 31 to 45 mins
- ◯ 46至60分鐘之間 Between 46 to 60 mins
- ◯ 1小時以上 1 hour more

* 6. 請問您 最近一個月內 都去哪裡 買生鮮雞蛋，大約 去幾次?

Have you purchased raw eggs in the following markets or stores in the last one month?

| | 沒有 0次 No, 0 time | 有, 1-2次 Yes, 1-2 times | 有, 3-4次 Yes, 3-4 times | 有, 5-6次 Yes, 5-6 times | 有, 7次以上 Yes, 7 times & up |
|---|---|---|---|---|---|
| 傳統市場 Traditional Market | ◯ | ◯ | ◯ | ◯ | ◯ |
| 農夫市集 Farmers' Market | ◯ | ◯ | ◯ | ◯ | ◯ |
| 超級市場 (全聯、頂好等) Supermarket (i.e. PX Mart, Welcome) | ◯ | ◯ | ◯ | ◯ | ◯ |
| 大賣場 (家樂福、大買家等) Hypermarket (i.e. Carrefour, Save & Safe) | ◯ | ◯ | ◯ | ◯ | ◯ |
| 會員制大賣場 (好市多) Hypermarket with a member fee (i.e. Costco) | ◯ | ◯ | ◯ | ◯ | ◯ |
| 食材專賣店 (里仁、主婦聯盟等) Organic specialty store (i.e. Leezen) | ◯ | ◯ | ◯ | ◯ | ◯ |
| 便利商店 (7-11、全家等) Convenience store (7-11, family mart) | ◯ | ◯ | ◯ | ◯ | ◯ |
| 網路商店購物 Online Shopping | ◯ | ◯ | ◯ | ◯ | ◯ |

# 雞蛋購買行為問卷調查 Eggs Purchasing Behavior Survey (Foods journal)

## 第1.2部分

*** 7. 請問您每次買生鮮雞蛋都花多少錢?**
**How much do spend when you buy raw eggs every time?**

- ◯ 20元以下 NT$ 20 or less
- ◯ 21 - 50元 NT$ 21 - 50
- ◯ 51 - 80 元 NT$ 51 - 80
- ◯ 81 - 110 元 NT$ 81 - 110
- ◯ 111 - 150 元 NT$ 111 - 150
- ◯ 151 - 170 元 NT$ 151 - 170
- ◯ 171 元以上 NT$ 171 or above

**8. 請問您買生鮮雞蛋至少都買多少顆?**
**Normally, how many raw eggs do you usually purchase each time?**

- ◯ 5 顆以下 5 eggs or less
- ◯ 6 - 8 顆 6 - 8 eggs
- ◯ 9 - 11 顆 9 - 11 eggs
- ◯ 12 - 14 顆 12 - 14 eggs
- ◯ 15 顆以上 15 eggs or more

*** 9. 下列哪些包裝標示項目會是您 一般 選購 生鮮雞蛋 的誘因? (可複選)**
**What items below will be your incentive to buy fresh raw eggs? (Check all that apply)**

- ☐ 有 溯源條碼 (QR code)
- ☐ 產銷履歷標章 Traceability label
- ☐ CAS 標章 CAS label
- ☐ 放牧蛋標章 Free range label
- ☐ 友善飼養標章 Friendly feeding label
- ☐ 動物福利標章 Animal welfare label
- ☐ 營養資訊 Nutrition information
- ☐ 品牌 Brand
- ☐ 特價 Special price
- ☐ 保鮮日期 Preservation date
- ☐ 符合食品安全管制系統 HACCP
- ☐ 有農夫的照片 Farmer picture
- ☐ 有藝人代言 Celebrity endorsement
- ☐ 雞蛋上有蓋印日期 A date stamp on eggs
- ☐ 洗選蛋 Clean washed egg
- ☐ 放山土雞蛋 Mountain chicken egg
- ☐ 我很少注意上述資訊 I seldom pay attention

# 雞蛋購買行為問卷調查 Eggs Purchasing Behavior Survey (Foods journal)

## 第2.1部分

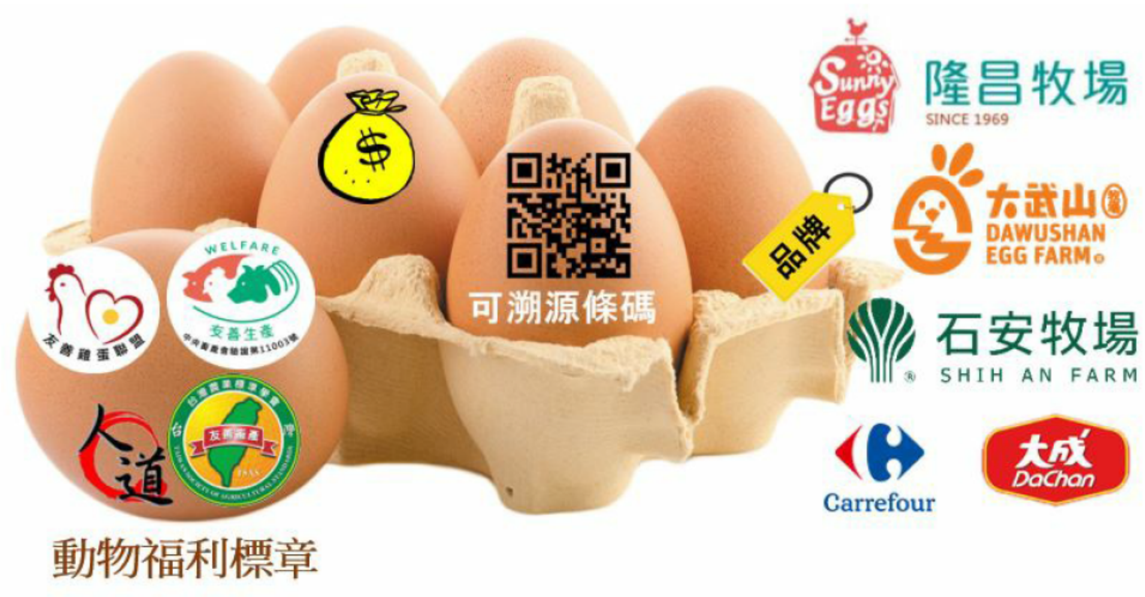


*** 10. 請問您曾在購物時掃過任何的QRcode嗎?**
**Have you scanned QR code when you purchase anything before?**

- ◯ 是，有掃過 Yes, I have scanned it before
- ◯ 沒有，沒有掃過 No, I have never scanned it before
- ◯ 不確定 Not sure

## 第2.2部分

*** 11. 請問您沒有掃過QRcode的原因是? (可複選)**
**Please tell us why you didn't scan QRcode before? (Check all that apply)**

- [ ] 包裝資訊已足夠，不須花時間掃條碼 Package information is enough, no need to scan QRcode
- [ ] 一點不感興趣 Not interested in it
- [ ] 沒注意到有QRcode可以掃 Not aware of QRcode
- [ ] 沒有多餘時間掃QRcode條碼 Don't have much time to scan during shopping
- [ ] QRcode的資訊我不太相信 Don't trust information in the QR code
- [ ] 我不常帶手機購物 I Seldom bring my cell phone during shopping
- [ ] 我害怕太複雜的資訊 I fear of the complex information
- [ ] 我的手機沒有綁網路 My cell phone don't have internet
- [ ] 其他

# 雞蛋購買行為問卷調查 Eggs Purchasing Behavior Survey (Foods journal)

## 第2.3部分

*** 12. 如果在 傳統市場 有提供 雞蛋 溯源條碼 QRcode ，請問您是否想掃看看?**
**Would you like to scan if the QRcode is provided in traditional markets?**

- ◯ 會的，如果我有時間的話 Yes, I'd like to when I have time
- ◯ 應該不會，即便不麻煩也是 Probably No, even it is no big trouble
- ◯ 不確定 Not sure

*** 13. 如果在 超級市場 有提供 雞蛋 溯源條碼 QRcode ，請問您是否想掃看看?**
**Would you like to scan if the QRcode is provided in Supermarkets?**

- ◯ 會的，如果我有時間的話 Yes, I'd like to when I have time
- ◯ 應該不會，即便不麻煩也是 Probably No, even it is no big trouble
- ◯ 不確定 Not sure

*** 14. 有關 雞蛋 的 溯源條碼 提供甚麼資訊時會促使你 想掃 QRcode? (至少勾選3項)**
**What information included in the QRcode would incentive you to scan? (At least choose 3 options)**

- ☐ 生產者資訊 (姓名、地址、聯繫電話) Producer's information (name, address, phone number)
- ☐ 產銷履歷達人介紹 The introduction of traceability experts
- ☐ 特價折扣碼 Discount code
- ☐ 推薦食譜 Recipe recommendation
- ☐ 生產認證標章 Production certificates label
- ☐ 檢驗資訊 Inspection information
- ☐ 其他, Others
- ☐ 詳細生產紀錄 Production record
- ☐ 詳細加工資訊 Processing Information
- ☐ 營養資訊參考 Nutrition Facts Reference
- ☐ 食物里程碳足跡 Carbon Footprint
- ☐ 蛋雞生產實境秀 Production live show
- ☐ 蛋雞生產影片回顧 Production video records

## 雞蛋購買行為問卷調查 Eggs Purchasing Behavior Survey (Foods journal)

### The last page

*** 15. 請問您的性別? What is your gender?**

- ◯ 男性 Male
- ◯ 女性 Female

*** 16. 請問您的出生年? What is your year of birth?**

[ ]

*** 17. 請問您的教育程度? Select the highest level of education you have achieved from the list below:**

- ◯ 國中(含)以下 Junior high school or below
- ◯ 高中職 High school
- ◯ 專科 Associate degree
- ◯ 大學 Bachelor's degree
- ◯ 研究所(含)以上 Graduate degree or above

*** 18. 請問您的職業？Which of the following best describes your employment status?**

- ◯ 已退休 Retired
- ◯ 待業中 Not employed
- ◯ 學生 Student
- ◯ 家管 Household
- ◯ 服務業 Service industry
- ◯ 軍公教 Government/Academic/Military
- ◯ 其它 Other (please specify)
- ◯ 製造業 Manufacturing
- ◯ 金融業 Financial industry
- ◯ 農林漁牧 Agriculture, forestry, animal
- ◯ 自由業 Freelancer
- ◯ 資訊科技業 Information technology industry
- ◯ 醫療與保健業 Medical and health care

[ ]

* 19. 請問您每月平均家庭收入大約多少？Please indicate the monthly family income in your family?

- NT$ 25,000 元以下
- NT$ 25,001 - 50,000 元
- NT$ 50,001 - 75,000 元
- NT$ 75,001 - 100,000 元
- NT$ 100,001 - 125,000 元
- NT$ 125,001 - 150,000 元
- NT$ 150,001 元以上

**20. 請問您家中有多少人同住? How many people are there in your household?**

- 只有我自己 One, only myself
- 2 人 2 people
- 3 人 3 people
- 4 人 4 people
- 5 人 5 people
- 6 人或以上 6 people or above

**21. 現在家中是否有未滿18歲的孩子同住? How many children under 18-year-old live with you?**

- 沒有 None
- 1 位小孩 1 child
- 2 位小孩 2 child
- 3 位小孩以上 3 children and more

**22. 請問您目前居住地點屬於? Which of the following best describes where you currently live?**

- 都市 City
- 郊區 Suburb
- 小城鎮 Small town
- 鄉村農村 Countryside (including farm)
- 山區 Mountain area

**23. 請問您住在哪個地區? Which area you are living at?**

- 北部地區 (基隆、台北、新北市、桃園、新竹)
  Northern area (Keelung, Taipei, New Taipei, Taoyuan, Hsinchu)
- 中部地區 (苗栗、台中、彰化、南投、雲林)
  Central area (Miaoli, Taichung, Chang Hua, Yunlin, Nantou)
- 南部地區 (嘉義、台南、高雄、屏東)
  Southern area (Chiayi, Taiwan, Kaohsiung, Pingtung)
- 東部地區 (宜蘭、花蓮、台東)
  Eastern area (Yilin, Hualian, Taitung)
- 其他地區 Other area.
